# Scalp reconstruction with locoregional and free flaps – a retrospective cohort study

**DOI:** 10.3205/iprs000187

**Published:** 2024-09-30

**Authors:** Olimpiu Bota, Franziska Beyer, Johann Klein, Tareq A. Juratli, Adrian Dragu, Kevin Bienger

**Affiliations:** 1 Department of Plastic Surgery, First Surgical Clinic, Emergency County Hospital Cluj-Napoca, Iuliu Haţieganu University of Medicine and Pharmacy , Cluj-Napoca, Romania; 2 University Center for Orthopedics, Trauma and Plastic Surgery, Faculty of Medicine Carl Gustav Carus, TU Dresden, Dresden, Germany; 3 Department of Neurosurgery, Medical Faculty and University Hospital Carl Gustav Carus, TU Dresden, Germany

**Keywords:** scalp reconstruction, plastic surgery, neurosurger, flap surgery, cranioplasty, infection

## Abstract

**Introduction:**

Scalp defect reconstruction requires interdisciplinary cooperation to restore soft tissue and osseous defects. While wound closure and form restoration, often a short-term treatment goal, ensures patient survival, the long-term preservation of the head and neck’s integrity and aesthetics is crucial for maintaining quality of life. This study aims to compare, quantify, and establish a safe and reproducible approach to various reconstruction options and the postoperative complication profile for individual scalp defect areas.

**Materials and methods:**

We retrospectively evaluated patients who underwent scalp reconstruction at our institution between March 2017 and April 2022. The inclusion criterion was the presence of a soft tissue defect at the cranium level.

**Results:**

We included 31 patients in the study (17 males, 14 females), with an average age of 61 years (range 17–92 years). Eight patients had received radiotherapy in the affected region. The mean defect size was 72.5±116 cm^2^ (range 20–441 cm^2^), and an average of 3±2 surgeries had been performed before the plastic surgical treatment was initiated. Eleven patients had only a soft tissue defect, while 20 patients had an associated bone defect. Fifteen of these patients received a cranioplasty. The rotation flap was the most frequently used (n=23), with or without split-thickness skin grafting, followed by the free latissimus dorsi muscle flap with split-thickness skin grafting (n=5), and the free lateral arm flap (n=2). Revision surgeries were necessary in 38.7% of cases due to wound healing disorders (n=9), bleeding (n=2), and cerebrospinal fluid leaks (n=1). Eventually, all wounds were successfully closed.

**Conclusion:**

Complex scalp defects can be closed using local flaps, thereby restoring aesthetics and tissue integrity. Free flaps remain a reliable solution for extensive defects. Moreover, in cases requiring cranioplasty, careful preoperative planning and an uncontaminated wound are essential for successful treatment.

## Zusammenfassung

**Einleitung:**

Die Wiederherstellung von Weichgewebe- und Knochendefekten bei der Rekonstruktion von Kopfhautdefekten erfordert eine interdisziplinäre Zusammenarbeit. Während der Wundverschluss und die Formwiederherstellung oft ein kurzfristiges Behandlungsziel darstellen, gewährleisten sie das Überleben des Patienten. Die langfristige Erhaltung der Integrität und Ästhetik des Kopfes und des Halses ist jedoch entscheidend für die Aufrechterhaltung der Lebensqualität.

**Material und Methoden:**

Wir haben Patienten retrospektiv ausgewertet, bei denen eine Kopfhautrekonstruktion zwischen März 2017 und April 2022 in unserer Einrichtung durchgeführt wurde. Das Einschlusskriterium war das Vorhandensein eines Weichteildefekts im Bereich des Kopfes.

**Ergebnisse:**

Wir nahmen 31 Patienten (17 Männer, 14 Frauen) in die Studie auf, mit einem Durchschnittsalter von 61 Jahren (17–92 Jahre). Acht Patienten erhielten eine Strahlentherapie in der betroffenen Region. Die durchschnittliche Defektgröße betrug 72,5±116 cm^2^ (20–441 cm^2^), und vor der plastisch-chirurgischen Rekonstruktion waren durchschnittlich 3±2 Operationen durchgeführt worden. Elf Patienten hatten nur einen Weichteildefekt, während 20 Patienten einen kombinierten Weichteil- und Knochendefekt aufwiesen. Fünfzehn dieser Patienten erhielten eine Kranioplastik. Am häufigsten wurde der Rotationslappen (n=23), mit oder ohne Spalthauttransplantation, gefolgt von der freien Latissimus-dorsi-Muskellappenplastik mit Spalthauttransplantation (n=5) und dem freien lateralen Oberarmlappen (n=2) verwendet. Revisionseingriffe waren in 38,7% der Fälle aufgrund von Wundheilungsstörungen (n=9), Blutungen (n=2) und Liquorlecks (n=1) erforderlich. Letztendlich wurden alle Wunden erfolgreich verschlossen.

**Schlussfolgerung:**

Komplexe Kopfhautdefekte können mit lokalen Lappen verschlossen werden, wodurch Ästhetik und Gewebsintegrität wiederhergestellt werden. Freie Lappen bleiben eine zuverlässige Lösung für ausgedehnte Defekte. Darüber hinaus sind in Fällen, die eine Kranioplastik erfordern, eine sorgfältige präoperative Planung und eine saubere Wunde für eine erfolgreiche Behandlung unerlässlich.

## Background

Scalp defects can result from trauma, infections, tumor extirpation, postoperative wound healing disorders, or radiotherapy. The goals of reconstruction are to restore form, function, and aesthetics of the region. While wound closure and form restoration remain primary concerns for ensuring patient survival, the function and aesthetic of the head and neck are crucial for preserving the quality of life in the long term [1].

The acronym SCALP conveniently summarizes scalp anatomy: skin, subcutaneous tissue, galea aponeurotica, loose connective tissue, and periosteum [2]. Defects in this region can be classified as superficial, leaving the periosteum intact; intermediate, exposing the cranial bone; or deep, where bone loss leaves the dura mater or substitute exposed. Restoration of skin and subcutaneous tissue is mandatory for all these defects. The Gillies and Millard principle, “replace like with like”, first stated in 1957, remains relevant in head and neck reconstruction today [3]. Consequently, the classical reconstructive ladder is often employed in scalp reconstruction, with split-thickness skin grafting and local and regional pedicled flaps playing crucial roles in contour restoration with hair-bearing or similar colour texture skin. For more complex and extended defects, free tissue transfers may be employed, particularly in scarred or irradiated wounds [4].

In deep scalp defects, the cranial bone must be replaced to protect cranial contents. Polymethylmethacrylate (PMMA) on-lays, titanium plates, computer-aided design (CAD) implants using Polyetheretherketone (PEEK), or autologous bone tissue can all be used to reconstruct the calvaria. Given that these are considered foreign bodies, a unique challenge presents due to biofilm formation and bacterial wound contamination. Cranioplasty is typically recommended three to six months after successful wound closure, although some authors report reimplantation as soon as two weeks post wound closure [5], [6], [7]. Scalp defects vary greatly in terms of their area, layers involved, and the patients’ wound healing capacity, resulting in a lack of standard procedures for defect coverage. 

This study aims to compare, quantify, and establish a safe and reproducible option for defect coverage, considering the variety of reconstruction options and the postoperative complication profile for individual defect areas of the scalp.

## Methods and statistics

We conducted a retrospective cohort study involving all scalp reconstructions carried out at our quaternary institution between October 2017 and April 2022. This study received approval from the institutional review board (BO-EK-266062022). The inclusion criterion was the presence of a soft tissue defect at the level of the cranium, encompassing the frontal, temporal, parietal, and occipital regions. We excluded wounds that were primarily closed without the need for reconstructive surgery. Data were retrospectively collected and organized using Excel 365 (Microsoft Corporation, WA, USA). Patient data included age, sex, cause of scalp defect, defect location and size, previous treatments, type of reconstructive procedure performed, and any postoperative complications.

Given the substantial variability among individual cases and a relatively small patient population, we determined that inferential statistics would not provide meaningful insights. Therefore, our analysis was confined to descriptive statistics, focusing on central tendencies (mean, median) and dispersion (range, standard deviation) for continuous variables, and frequencies for categorical variables.

## Results

**Figure 1:**
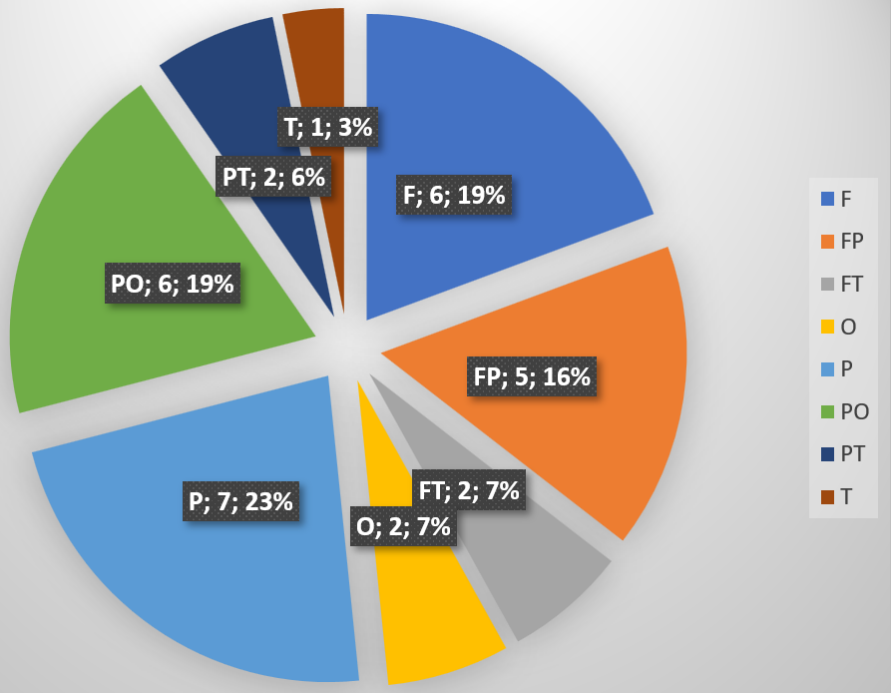
Anatomical distribution of the wounds F – frontal, FP – frontoparietal, FT – frontotemporal, O – occipital, P – parietal, PO – parietooccipital, PT – parietotemporal, T – temporal

**Figure 2:**
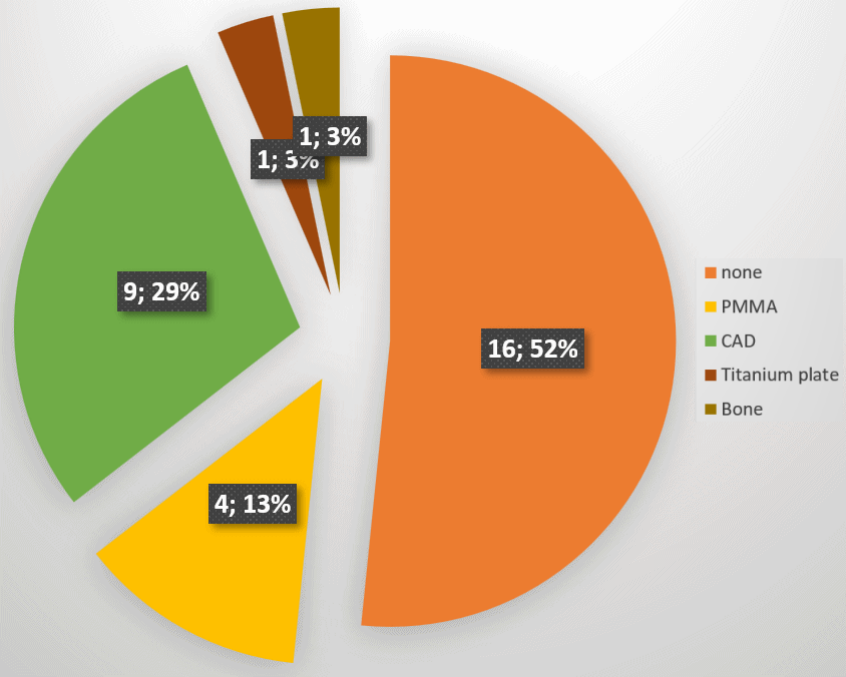
Bone reconstruction techniques CAD – computer-assisted design, PMMA – Polymethylmethacrylate

We included thirty-one patients in the study, comprised of 14 females and 17 males. The average age of the patients was 61 years (range 17–92 years). Sixteen patients had received neurosurgical treatment, one patient had been treated in the otorhinolaryngology department, while the remaining patients had been treated exclusively in the plastic surgery department. The etiology was tumoral in 21 patients, traumatic in five, and five patients had non-traumatic insults to the brain which required neurosurgical intervention. All cranial regions were affected (Figure 1 (Fig. 1)). Eight patients had received radiotherapy in the affected region. The mean defect size was 72.5±116 cm² (range 20–441 cm²) and on average, 3±2 surgeries had been performed before plastic surgical treatment was initiated (Table 1 (Tab. 1)). Eleven patients had only a soft tissue defect, while 20 patients had an associated bone defect. Among these, 15 patients received cranioplasty (Figure 2 (Fig. 2)). Table 2 (Tab. 2) provides an overview of the tumoral cases.

**Figure 3:**
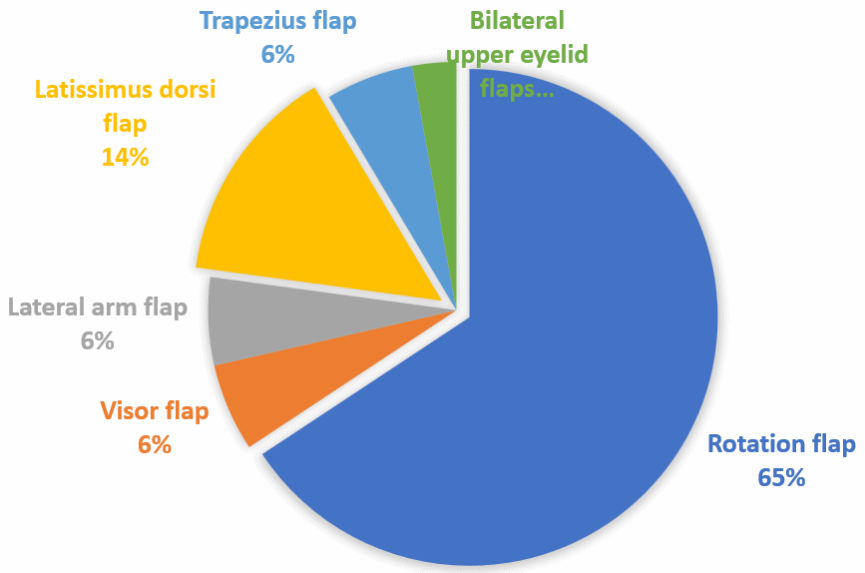
Types of flap coverage

Reconstructive treatment was accomplished within a single surgery in four cases, while in 27 patients the wound was temporarily covered with a synthetic skin substitute or with negative pressure wound therapy (NPWT). Thirty-five flaps were performed in 31 patients. The most frequently used flap was the rotation flap with or without split-thickness skin grafting, followed by the free latissimus dorsi muscle flap with split-thickness skin grafting, the free lateral arm flap (LAF), the local visor flap, and the trapezius musculocutaneous flap (Figure 3 (Fig. 3)). Twelve patients developed a complication and required revision surgery. These complications included nine instances of wound-healing disorders, two postoperative bleedings, and one cerebrospinal fluid leak. Eventually, all wounds were successfully closed.

**Figure 4:**
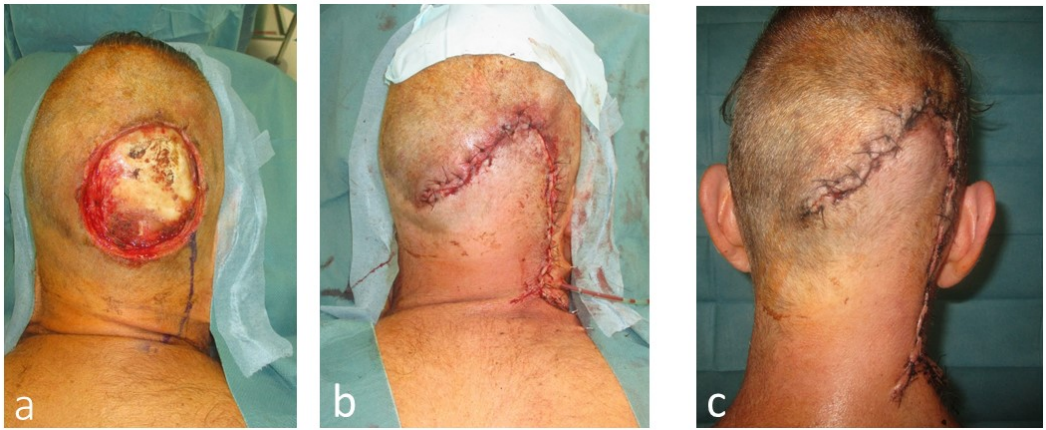
Case 1: 50-year-old male with 150 cm^2^ defect with the exposed occipital bone after resection of a Dermatofibrosarcoma protuberans a: 150 cm^2^ defect with exposed occipital bone. b: Wound closed with an occipital rotational flap. c: Finding 7 days postoperative.

**Figure 5:**
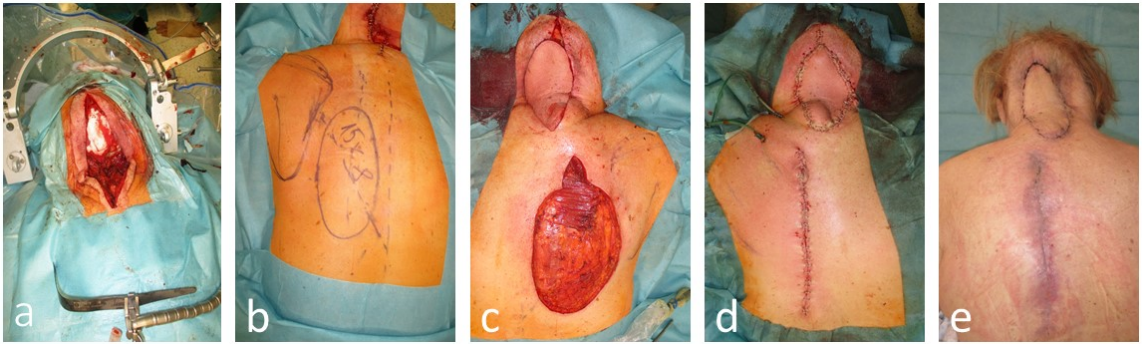
Case 2: 65-year-old female with a 10 cm^2^ occipital defect with exposed dura mater after resection of an ovarian carcinoma metastasis a: 10 cm^2^ occipital defect with exposed dura mater. b: Design of a pedicled lower trapezius musculocutaneous flap. C: Flap transferred before donor site closing. D: Final intraoperative result. e: Finding 14 days postoperative.

**Figure 6:**
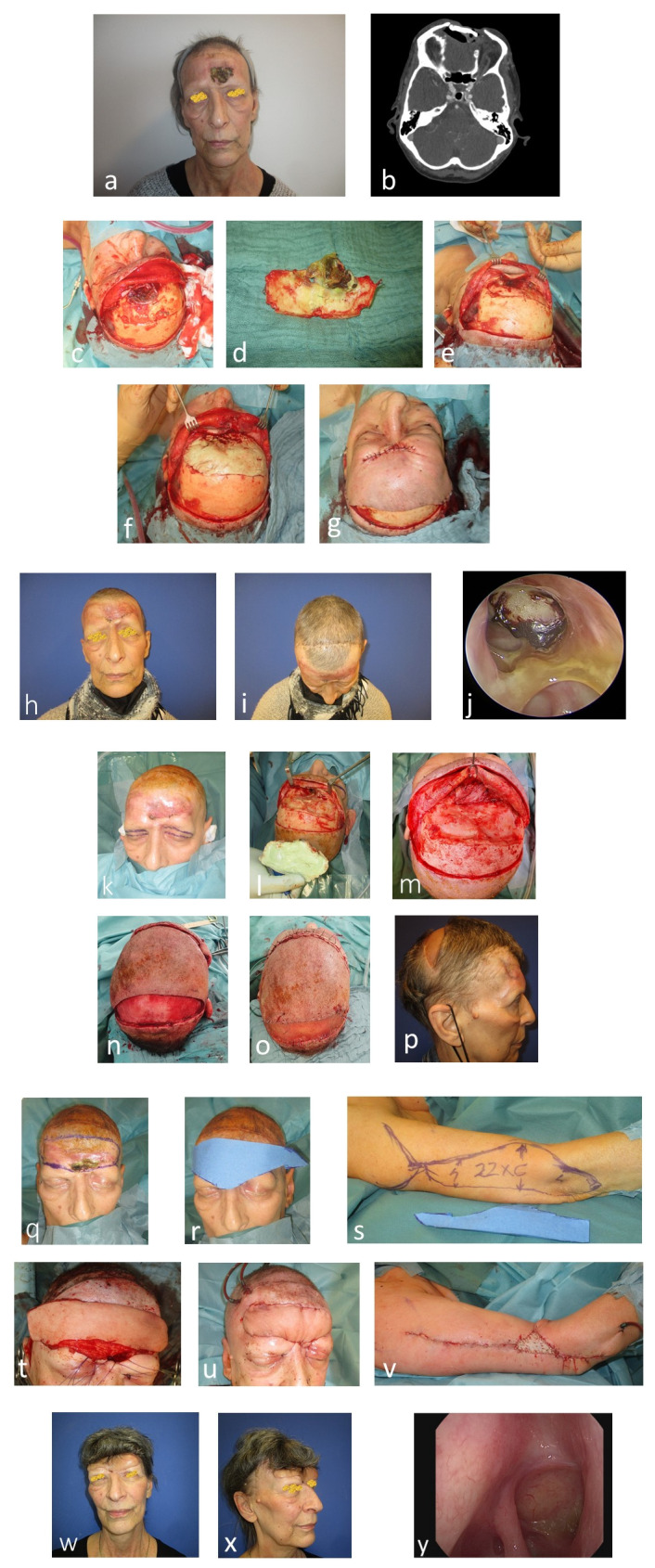
Case 3: 69-year-old female with a frontal soft tissue and bone defect after resection of a high-grade squamous cell carcinoma of the frontal sinus and filling with PMMA spacer a: Frontal soft tissue and bone defect. b: CT scan. c: Intraoperative finding. d: Resected necrotic frontal bone together with the PMMA spacer. e: Communication with the nasal cavity. f: Formed PMMA spacer with Vancomycin. g: Wound closure with visor flap and parietal undermining. h: Postoperative anterior view. i: Postoperative parietal view. j: Endoscopic view of the PMMA spacer. k: Second surgery. Planning for bilateral upper lid flaps to close the nasal cavity. l: Removal of PMMA spacer. m: Transplanted bilateral upper lid flaps. n: Visor flap. o: Split thickness skin grafting of the donor site. p: Postoperative side view. q: Skin necrosis frontal. r: Template of the planned flap with the removal of the whole frontal aesthetic unit. s: Lateral arm flap before harvesting. t: Transplanted flap; the lower part was deepithelized and turned over to close the nasal cavity. u: Sutured flap; no spacer was used at this surgery. v: Donor site. w: Postoperative anterior view six months postoperative. x: Postoperative side view six months postoperative. y: Endoscopic view with closed communication to the frontal region.

**Figure 7:**
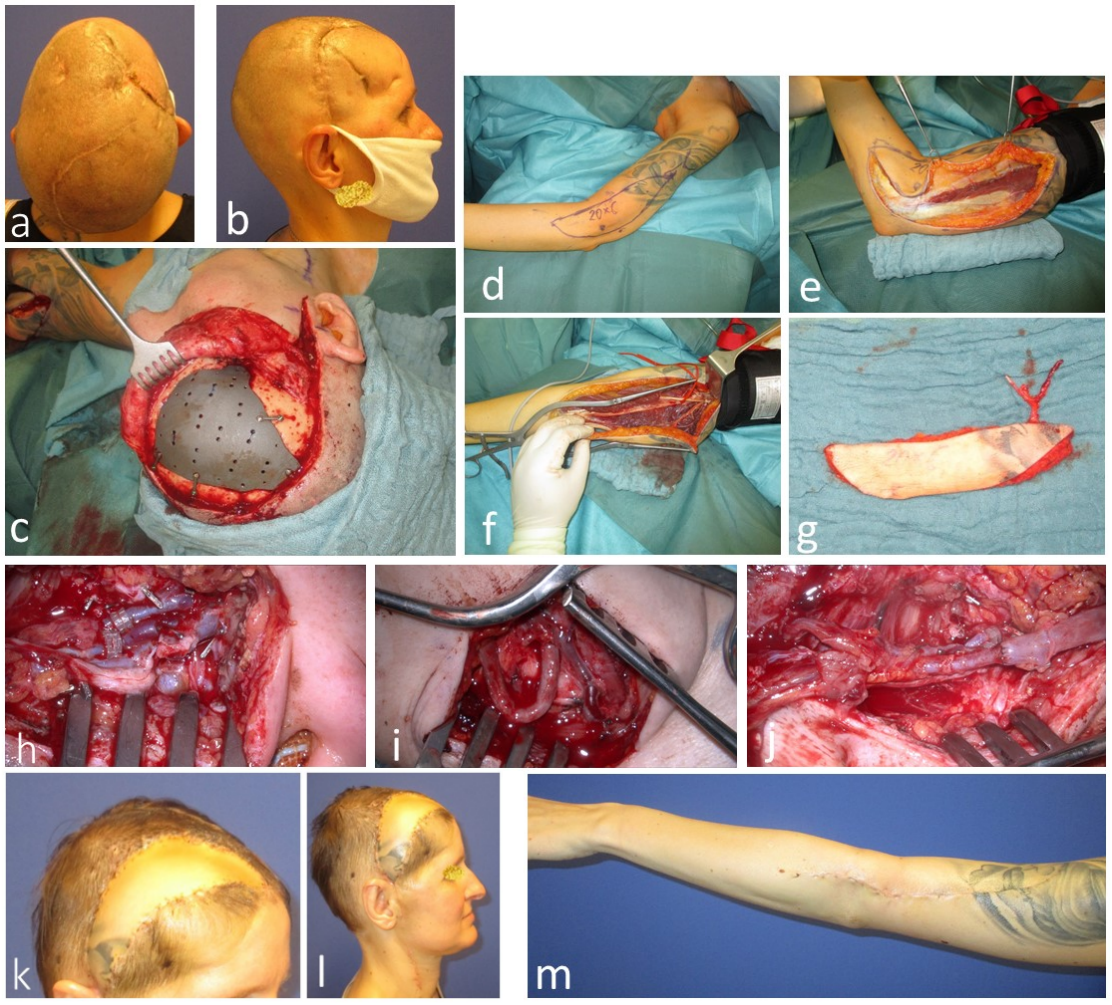
Case 4: 39-year-old female with extended scaring of the frontotemporal region after trepanation 20 years ago with osteonecrosis and multiple failed cranioplasties a: Preoperative cranial view. b: Preoperative side view. c: Cranial bone reconstruction with titanium plate. d: Design of extended lateral arm flap. e: Flap harvesting from lateral. f: Flap harvesting from the medial. g: Harvested flap. h: Intraoperative thrombosis of the superficial temporal artery. i: Vena saphena magna graft, anastomosed to the superior thyroid artery. j: Cranial anastomosis of the graft to the flap artery (deep brachial artery). k: Postoperative anterolateral view. l: Postoperative lateral view. m: Donor site.

**Figure 8:**
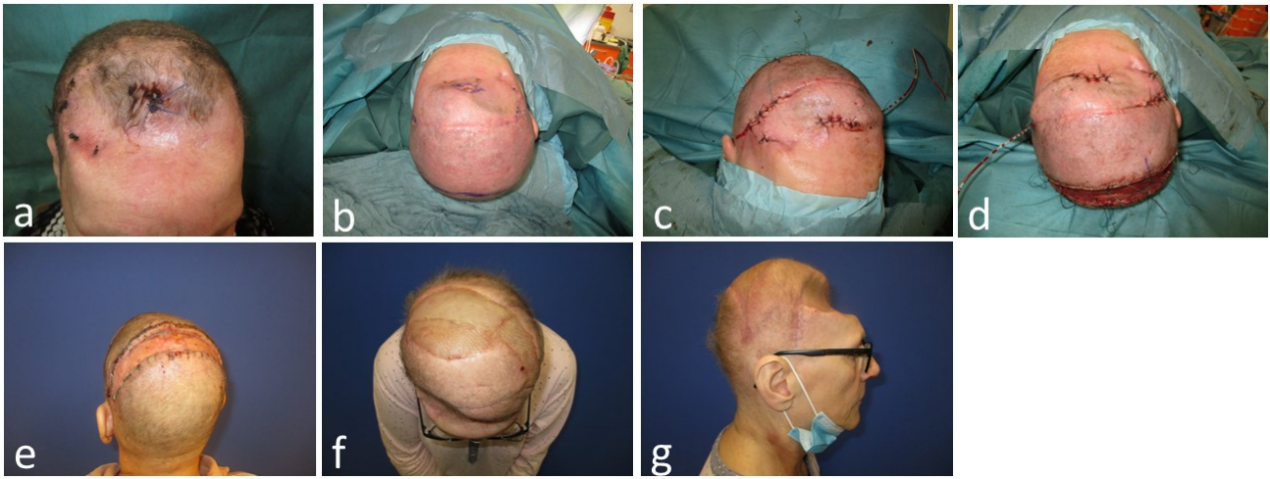
Case 5: 65-year-old female with cranial defect and multiple frontal wound healing disorders after resection of brain metastasis of an invasive ductal carcinoma. Four revisional surgeries with repeated cranioplasties were performed before the patient was referred to plastic surgery. a: Initial finding with multiple wound healing disorders in the frontal and temporal regions. b: Planning of the visor flap to augment the frontal soft tissues and debride and close the wounds. c: Frontal region after wound closure. d: Visor flap and donor site covered with split-thickness skin grafting. e: Donor site seven days postoperative. Notice the insufficient periosteum around the midline anteriorly due to the previous harvest for duraplasty. The split-thickness skin graft was lost in this region. f: Result six weeks after free latissimus dorsi flap coverage with split-thickness skin grafting, anterolateral view. g: Result six weeks after free latissimus dorsi flap coverage with split-thickness skin grafting; lateral view.

In the following section, we present five case reports from this cohort (Figure 4 (Fig. 4), Figure 5 (Fig. 5), Figure 6 (Fig. 6), Figure 7 (Fig. 7), Figure 8 (Fig. 8)).

## Discussion

The restoration of skull integrity, functionality, and aesthetics after cranioplasty necessitates effective soft tissue reconstruction. This process is crucial to mitigate the risks of infection and implant exposure. While advanced reconstructive surgery strategies prioritize complex distant flap techniques such as free flaps, scalp reconstruction largely remains reliant on the traditional reconstructive ladder [1]. This is due to the fact that the best aesthetic and functional results, including the restoration of hair-bearing skin, are most effectively achieved using local tissues. Techniques such as secondary healing, with or without the assistance of negative pressure wound therapy, present a simplistic approach to wound closure in cases lacking a cranial bone defect [2]. These methods facilitate wound and scar contraction, yielding an aesthetically satisfactory result, particularly in bald men. Dermal substitutes, like Integra (IDRT, Integra LifeSciences, Princeton, NJ, USA) or Matriderm (MedSkin Solutions Dr. Suwelack AG, Germany), have also been used successfully, typically after burring of the external cortical bone to promote granulation tissue ingrowth [3], [4]. This results in a well-vascularized, stable substrate which can be split-thickness grafted.

Direct wound closure in small defects can be assisted by galea scoring, allowing for a mild stretch of the skin and subcutaneous tissue. Local flaps, encompassing rotation, transposition, and advancement flaps, are of paramount importance in scalp reconstruction [3], [5], [6]. Through redistribution of local tissues, prompt reconstruction with hair-bearing tissues can be achieved. In more extensive defects, the flap can be elevated from the opposite healthy side and the donor site grafted with split-thickness skin [2]. 

Free flaps are also important in scalp reconstruction, particularly when extensive scarring, irradiation, or large defects preclude wound closure with local tissues [7]. Though skin from remote regions may vary in color, texture, and pilosity from scalp skin, free flaps, such as the latissimus dorsi muscle flap, provide an adequate solution for reconstructing large scalp defects [8]. Alternatively, the fasciocutaneous flaps can offer adequate soft tissue coverage with minimal donor site morbidity. While the anterolateral thigh flap is the workhorse flap for plastic surgery, its different skin texture and bulkiness may offer suboptimal results [9], [10]. The lateral arm flap is an adequate solution for covering medium-sized soft tissue defects. Its reduced bulkiness, especially in the distal part, is a good match to the scalp and the skin color, although different from the face, is less conspicuous than the skin from the abdomen or lower extremity. For aesthetic improvement, hair transplantation can be considered after wound healing.

Cranioplasty, the process of cranial bone reconstruction post-craniotomy or craniectomy, calls for an implant that meets several criteria: brain protection, biocompatibility, infection resistance, malleability, radiolucence, thermal neutrality, affordability, and accessibility [11]. Currently, no such perfect implant exists. Common practice entails storage of the removed bone flap post-decompressive craniotomy by dry freezing or preserving in the patient's abdominal fat [11]. In situations where this is not viable, such as oncological cases (case 3) or incidents of traumatic bone destruction, infection, or osteonecrosis (case 4), alternatives like bone autografts have been employed. These alternatives, however, introduce the disadvantage of donor site morbidity [11]. 

Various polymers have been used in cranial reconstruction, including celluloids, hydroxyapatite, polyethylene, ceramic, PMMA, and PEEK [12], [13], [14]. PMMA is one of the most common materials, often used in conjunction with titanium plates and affixed to the surrounding cranium with plates and screws. Despite its higher cost, computer-aided designs using PEEK have gained significant ground in cranioplasty due to their improved biocompatibility and form [15]. Among various metals used in cranioplasty including gold, platinum, silver, tantalum, and aluminum, titanium meshes are the most preferred due to their biocompatibility and resistance to mechanical forces and infection [13]. A comprehensive review of cranioplasties revealed a 14% overall surgical revision rate, which can be further reduced using indocyanine green video angiography [16]. PEEK implants showed the lowest reoperation rate, trailed by titanium, hand-formed PMMA, hydroxyapatite, preformed PMMA implants, and autografts. The rate of implant exposure was 6%, while infection occurred in 8% of cases [14]. 

Cranioplasty has been established as a procedure that enhances cerebral function, protection, aesthetics, and overall quality of life [11], [17]. In instances where infection or implant exposure occurs, a biofilm is likely to develop around the implant [18]. Traditional treatment protocols suggest implant removal and re-implantation after a span of three to six months [11], [19]. However, contemporary approaches favor retaining the implant in early infections, single-stage revision and implant exchange, or early re-implantation two weeks post-removal, accompanied by a 12-week course of antibiotics. This method is aimed at minimizing the functional and aesthetic impact tied to the loss of cranial protection [20], [21]. In our study, 20 patients exhibited cranial bone defects. A significant majority had already undergone multiple revision surgeries due to recurrent infections, averaging 3±2 surgeries prior to being referred for plastic surgery. This trend rendered cranial bone reconstruction infeasible for five patients due to the high probability of persistent biofilm in the wound, and the consequent risk of infection recurrence.

Our study cohort covered a wide range of etiologies, locations, defect sizes, and locations around the calvaria. Our findings reinforce the effectiveness of careful planning and execution in the reconstructive process, utilizing the reconstructive ladder in an interdisciplinary setting for successful wound closure. Factors such as extended scarring, radiotherapy, osteomyelitis, wound contamination, biofilm, and the patient's oncological status must be considered in the treatment of these defects. The five highlighted cases illustrate the complexity of treatment for these patients and underscore the critical role of judicious selection of reconstructive options.

The first case (Figure 4 (Fig. 4)) features a young, active patient with an extended occipital defect and exposed occipital bone. A fast wound closure with a hair-bearing approach was the preferred choice, adhering to the principles of “stealing from Peter to pay Paul” and “learning to control tension” [22]. The second case (Figure 5 (Fig. 5)) represents an oncological palliative situation, with a terminally ill patient requiring a swift and robust coverage of the exposed occipital dura mater. In this instance, aesthetics were not a priority, and a caudal musculocutaneous trapezius flap was the optimal choice given the patient’s overall condition.

The third case (Figure 6 (Fig. 6)) details the complex resection of a high-grade squamous cell carcinoma in the frontal sinus. This required the removal of the foreign body (PMMA spacer) and the contaminated frontal bone. Following this, closure was performed using an advancement flap with galea scoring. Subsequent complications arose due to communication between the nasal cavity and the newly implanted spacer. To address this, surplus tissue from the upper eyelids was used to seal the connection to the nose. Despite these measures, the spacer in the nose cavity was exposed, leading to re-contamination with multi-drug resistant *Pseudomonas aeruginosa* (PA). This necessitated the use of a free flap. A LAF was designed to reconstruct the entire aesthetic unit of the forehead (Figure 6r,s (Fig. 6)), and a section of the flap was used to close the nasal cavity. Following deliberation, the implantation of a PMMA spacer was forgone, resulting in successful wound healing and a closed nasal cavity (Figure 6y (Fig. 6)). The final aesthetic outcome was satisfactory, despite a slight skin color discrepancy between the forearm flap and the facial skin. The patient was satisfied with the result and declined further cranioplasty.

Case four (Figure 7 (Fig. 7)) involves a 39-year-old female with late osteonecrosis following trepanation 20 years prior. She underwent six revision surgeries, leaving her scalp extensively scarred. To enable a new cranioplasty, a free LAF flap was planned, as the thin tissue matched well with the scalp skin and provided ample coverage for the titanium cranioplasty. Hair transplantation was also considered. Intraoperatively, it was observed that the temporal vessels, intended for anastomosis, were occluded due to previous surgeries. The vessels were dissected down to the zygomatic arch, where blood flow was adequate for arterial and venous anastomosis. However, an early thrombosis of the temporal artery was encountered. As a workaround, a great saphenous vein graft was used to connect the flap artery to the superior thyroid artery in the neck (Figure 7 i,j (Fig. 7)). The final outcome was satisfactory, and the patient chose to delay the hair transplantation (Figure 7 k,l (Fig. 7)).

Lastly, the fifth case (Figure 8 (Fig. 8)) presents a 65-year-old female with a frontal cranial defect and wound healing disorders after breast cancer metastasis resection (Figure 8a (Fig. 8)). A visor flap was designed and used to augment the frontal skin, to excise and close the wounds. Although an appropriate flap width was designed, the periosteum had been harvested back to the flap incision line during the neurosurgical intervention. Postoperative there was a small loss of the split-thickness skin grafting at the donor site due to the insufficient periosteum. The wound failed to heal and a free latissimus dorsi flap was designed and transplanted to close the parietal defect, with vessel anastomosis to the temporal artery and vein.

In conclusion, while scalp reconstruction and cranioplasty present significant challenges, judicious selection of reconstructive strategies can yield successful outcomes. An interdisciplinary approach is crucial in ensuring that all factors are taken into account in the management of such complex cases.

## Notes

### Ethics approval and consent to participate 

Institutional Review Board (IRB) Approval: Approval from the Ethics Committee of TU Dresden was obtained (BO-EK-266062022). An informed consent to participate was waived by the Ethics Committee of TU Dresden. All methods were carried out by the guidelines and regulations. 

### Consent to publish 

The patients gave their consent for publication.

### Competing interests 

The authors declare that they have no competing interests.

### Authors’ contributions 

OB conceptualized the study, wrote and edited the manuscript. KB gathered the data. FB performed the statistical analysis. JK, TAJ and AD substantially revised the final version of the manuscript. All authors read and approved the manuscript. 

### Acknowledgments 

Bianka Herzog helped with the patient examination and photo acquisition.

## Figures and Tables

**Table 1:**
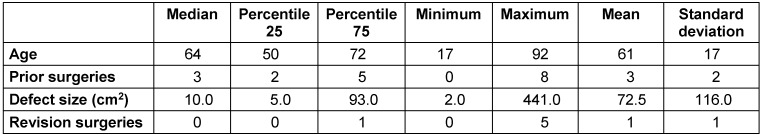
Operations classification before plastic surgery treatment

**Table 2:**
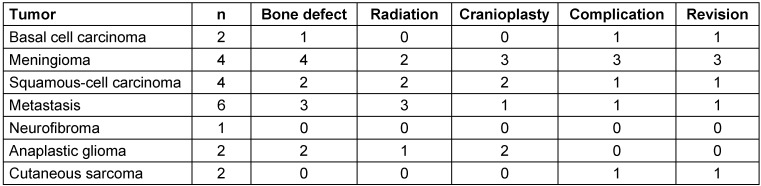
Classification and subsequent therapy of the individual tumor types
